# Sedentary behavior among university students and its socio-ecological correlates during the academic day: a cross-sectional study

**DOI:** 10.3389/fpubh.2026.1754818

**Published:** 2026-08-17

**Authors:** Juliane Möckel, Birgit Wallmann-Sperlich, Robert Rupp, Jens Bucksch

**Affiliations:** 1 Department of Prevention and Health Promotion, Heidelberg University of Education, Heidelberg, Germany; 2 Integrative and Experimental Exercise Science and Training, Faculty of Human Science, Institute for Sports Science, Julius-Maximilians University of Würzburg, Würzburg, Germany

**Keywords:** academic setting, higher education, physically active learning, sitting, socio-ecological model, teaching units, university students, university teaching

## Abstract

**Background:**

Prolonged sitting is linked to adverse health outcomes and is particularly prevalent among university students. While individual determinants have been widely studied, less is known about the broader range of socio-ecological influences on sitting during structured academic activities. This study aims to examine the prevalence of sedentary time and its socio-ecological correlates within the academic course of the day of university students.

**Methods:**

A cross-sectional online survey was conducted among 434 students (81.8% female) with a mean age of 22.9 ± 3.5 years. Participants self-reported their sedentary time during the academic day and within 90-min teaching units, using an adapted version of the Occupational Sitting and Physical Activity Questionnaire (OSPAQ). Correlates at individual, social and environmental levels were also self-reported. Logistic regression models were used to identify associations with (1) overall sedentary time, (2) uninterrupted sitting during 90-min teaching unit, and (3) number of sitting breaks during 90-min teaching units.

**Results:**

Students self-reported sitting for an average of 12.4 ± 3.2 h per day, with 55% taking no breaks during lectures. Motivational self-efficacy and autonomy were associated with reduced sitting and more frequent interruptions of sitting. Social norms supporting movement predicted less sitting, while subjective norms encouraging sitting were linked to more sedentary patterns. A self-reported movement-supportive environment showed strong associations with sitting interruptions during lectures, both in bivariate [OR = 3.43, 95% CI (1.98–5.95)] and multivariate analyses [OR = 2.14, 95% CI (1.04–4.40)].

**Conclusion:**

Our findings demonstrate that sedentary behavior among university students is shaped by factors across multiple socio-ecological levels. Motivational self-efficacy emerged as the most consistent individual-level correlate, significantly associated with all three sitting outcomes. These findings highlight the need for interventions that address both individual correlates and the structural embedding of sitting in the university context at a social and environmental level. Universities should consider reforms that reduce sedentary constraints and expand behavioral options, such as integrating physically active teaching formats and redesigning learning spaces.

## Introduction

Prolonged sitting has been linked to adverse physical and mental health outcomes, including musculoskeletal complaints, metabolic risk factors and reduced wellbeing (1). These issues are particularly relevant for university students, who have been identified as a high-risk group for sedentary behavior (SB) due to their academic environment characterized by long lectures, extensive computer use and limited structured physical activity (PA) (2, 3). As an example of the economic burden of sitting, sitting-related cancer costs in Germany were estimated at approximately € 797 million in 2024 (4). Despite efforts to reduce sedentary time, detailed knowledge about the contexts and determinants of sitting in higher education settings remains limited. Understanding when, where and why sitting accumulates among students is essential for developing effective, targeted interventions in this key stage of life.

Although young adults are generally considered healthy, university students have been identified as a group with conditions which highly support being sedentary. Over the past few decades, university students have shown the steepest increase in sitting and the most pronounced decline in moderate-to-vigorous PA compared to other age groups (5–7). For example, moderate-to-vigorous PA levels drop from an average of 26 min per day in the first semester to 18 min in the final year, and one in three students report becoming less active and more sedentary after entering university (5). Since behaviors established in early adulthood tend to persist (8), prolonged sedentary time during this period may have long-term implications for individual and population health. Moreover, university students represent a growing share of the young adult population in high-income countries, with substantial influence on future societal norms and health behaviors (9, 10). Recent Organization for Economic Co-operation and Development data show that around 54% of individuals aged 18–24 years are currently engaged in education or training and, 43.1% of adults aged 25–34 years across the European Union already hold a tertiary qualification (11, 12). In Germany, approximately 2.87 million people are enrolled at universities in the winter term 2024/25, underlining the considerable size of this subpopulation (13).

According to the World Health Organization (WHO) 2020 guidelines on PA and SB, all adults should limit sedentary time and interrupt prolonged sedentary times every 30–60 min to reduce associated health risks (14). Prolonged and uninterrupted sedentary times are a core issue of academic student life. The proportion of students sitting for more than 8.5 h daily is 17%, which is only surpassed by ‘other white collar workers’ at 19% and equals that of managers (15). Studies report average sitting durations of up to 14.4 h per day (2), well above the global average of 4.7 h (16). However, the prevalence varies considerably depending on the assessment methods, with higher prevalence typically reported when objective measures are used (17).

To better understand sitting among university students, it is essential to distinguish where and how sitting occurs across the full day, during the academic day and within individual teaching units. At the whole-day level, sedentary time includes not only academic activities but also transportation, meals and screen-based leisure, such as watching television, browsing social media or gaming (18–20). Passive commuting by car or public transport further adds to daily sedentary time (2, 21). Within the university context, sedentary time is mainly composed of academic tasks. Students spend prolonged periods of sitting in lectures, seminars and during individual study sessions (10, 20, 22). In the context of the present study, the academic day refers to all sedentary periods associated with university-related activities, including both structured class time (e.g., lectures, seminars) and individual study periods (e.g., reading, writing, computer-based assignments). Time spent in screen-based leisure activities such as gaming or social media use was not considered part of the academic day. These academic tasks and phases are cognitively demanding, but are mostly executed by being physically inactive, making them particularly relevant for targeted interventions. Zooming in further, individual 90-min teaching units are typically characterized by uninterrupted sitting, with limited opportunities for breaking up sitting and being physically active.

An increasing number of studies highlight that not only the total amount of sedentary time but also its pattern is crucial: prolonged, uninterrupted sitting poses greater health risks than sitting that is regularly interrupted (23–26). So-called micro-breaks such as standing, stretching, or walking a few steps can mitigate adverse effects and may be particularly feasible in educational contexts (27–29).

To understand why students remain sedentary despite these risks, the socio-ecological model provides a useful framework for analyzing behavior across multiple, interacting levels (30, 31). The model highlights that sitting is shaped not only by individual factors such as habits, attitudes and self-efficacy, but also by social norms, physical environments and institutional structures. In this study, we apply this framework to identify determinants of sitting at the individual, social and environmental levels within higher education settings. At the individual level sitting among students is influenced by, e.g., self-efficacy to change these behaviors and perceived autonomy (32, 33). High self-efficacy regarding the reduction of sitting is commonly reported among students; however, this does not always lead to a behavioral change, as perceived distant health risks and structural barriers, such as rigid class schedules and limited environmental support, often inhibit action (34).

On the social level, norms and peer expectations influence behavior. The expectation to remain still and avoid distracting others can discourage being physically active, especially in group learning contexts (35). This is in line with findings by Rupp et al. (29), who identified strong organizational sedentary norms among lecturers and the anticipation that students expect to sit as major social barriers to the implementation of physically active teaching. Both subjective norms, referring to perceived expectations from significant others, and broader social norms, capturing what is commonly accepted or practiced within a group, have been identified as relevant determinants of sitting (8, 34). Current evidence suggests that stronger subjective norms in favor of movement are associated with greater intentions to reduce sedentary time (8), while prevailing social norms in academic settings often reinforce prolonged sitting as the default mode of conduct (29).

At the environmental level, physical features such as furniture design, spatial constraints and absence of activating resources (e.g., standing desks) play an important role (36). In addition, structural aspects of the learning environment, such as fixed seating arrangements, limited classroom space and lack of opportunities for movement, can further reinforce sitting. For example, institutional layouts that do not support posture variation or movement have been found to restrict students’ ability to interrupt prolonged sitting (37). Institutional structures further compound these environmental factors. Rigid class schedules, traditional classroom setups and implicit expectations within academic settings often limit students’ autonomy to change postures or take breaks during lectures. Such constraints can reinforce prolonged sitting and hinder efforts to reduce sitting (38, 39).

This study follows an exploratory approach to examine the prevalence of sitting in academic contexts and better understand the socio-ecological determinants of sitting in university settings. While existing research has examined overall sedentary time among students, less attention has been paid to their behavior during structured academic sessions. Addressing this gap, the study pursues three objectives:

1. To assess the prevalence and duration of SB among university students, with particular attention to the sedentary time accumulated throughout the academic course of the day.
2. To examine the extent to which students are sedentary and how often they take breaks from sitting during typical 90-min teaching units.
3. To identify individual, social and environmental correlates of overall sedentary behavior and the interruption of sedentary time within academic settings.

## Methods

### Study design and setting

This cross-sectional study collected data using a self-reported questionnaire (see Supplementary file 1), which was distributed online using LimeSurvey. The questionnaire was developed based on validated and internationally used instruments (see Table 1), covering constructs relevant to the socio-ecological approach. The questionnaire was pretested with 18 participants to assess comprehensibility and completion time with a structured written protocol using a think aloud technique. The results led to minor wording adjustments and changes to response formats (e.g., the addition of examples for light, moderate and vigorous PA or the conversion of an item into a filter question).

**Table 1:** Summary of independent variables and measurement details.

Socio ecological level	Independent variables/construct	Description	Number of items (Cronbach’s α*)	Example item	References
Individual	Habitual sedentary behavior	Unconscious sedentary behavior during lectures.	1	*“Sitting is something I do in class without thinking about it.”*	(32, 36, 38)
	Cue-dependent breaks from sitting	Need for external prompts to interrupt sitting.	1	*“I need a prompt/reminder to remember to interrupt sitting more often in class.”*	(32, 36, 38)
	Motivational self-efficacy	Motivational self-efficacy to reduce sitting.	5 (0.748)	“I am confident that I can regularly interrupt my sitting time during classes.”	(33)
	Perceived autonomy	Perceived autonomy in deciding when and how long to sit.	2 (0.818)	“I am allowed to decide for myself when I stand up during class.”	(32, 36, 38)
	Attitude toward reducing sitting	Attitude toward reducing sitting during lectures.	2 (0.841)	*“I believe prolonged, uninterrupted sitting negatively affects my health.”*	(36)
	Mental state	Indicators of students’ mental state during lectures, e.g., restlessness, fatigue, declining concentration and motivation.	5 (0.856)	“Decreasing ability to concentrate”	(25)
	Physical wellbeing	Physical wellbeing regarding sitting-related discomfort during lectures.	4 (0.683)	“Tension in the neck and shoulder area”	(25)
Social	Perceived social norm	Perceived cultural support for interrupting sitting within the academic environment.	3 (0.832)	“In the classes I attend, there is a culture that supports standing and movement.”	(36)
	Subjective norm	Reflecting perceived approval by others to break up sitting.	2 (0.772)	“I worry that I might be perceived as inattentive if I sit less during classes.”	(36)
Physical environment	Perceived physical environment	Perceived physical environment supporting active behavior.	4 (0.740)	“Do you have access to sit-stand furniture (e.g., standing desks, desk risers) during classes?”	(36)

*Calculated based on the present dataset and for the summarized scales and composite measures.

Completing the section of the questionnaire relevant to this article took approximately 20 min; however, as data were also collected for an associated subproject, the total duration increased accordingly to up to 45 min on average.

The study was conducted at Heidelberg University of Education. A full census was targeted by inviting all students at the university to participate. No exclusion criteria were applied, as all students were eligible to participate regardless of their physical condition, health status or disability. Both undergraduate and postgraduate students participated in the study. At the latest count in 2023, the total on-campus student population at the institution was 5,050. Data collection took place over 3 weeks during the winter semester of 2023/24. Recruitment efforts included university-wide emails, social media posts, flyers, electronic newsletters, and word-of-mouth communication, resulting in a sample of 822. The response rate was 16.3%. This figure includes cases with missing data; a detailed account of missing values is provided below. Participation in the study was voluntary, and informed consent was obtained from all participants before the start of the survey. Ethical approval for the study was granted by the Joint Ethics Committee of the Heidelberg University of Education and the SRH University of Applied Sciences Heidelberg (approval code: EV2023_07).

### Variables

The following section provides an overview of the variables used in the analysis, beginning with the outcome variables, followed by the independent variables and potential covariates.

### Outcome variables

In order to provide a comprehensive assessment, the following outcome variables were examined.

#### Outcome 1: proportion of daily time spent sitting

Sedentary time per day was assessed using a single self-report item. Participants were asked to estimate the percentage of their study-related time spent sitting, standing, walking or engaging in vigorous PA over the past 7 days (total = 100%). Study-related time included time in classes, self-directed study at home or in the library and other study-related tasks. Commuting and leisure-time activities were explicitly excluded. The proportion of sedentary time (%) was used as the outcome variable. Based on the sitting proportion, a dichotomous variable was constructed using the sample median (80%) as the cutoff, as no validated population-specific threshold exists for this outcome measure and the distribution was right-skewed. Participants reporting more than 80% sedentary time were used as the reference group. This item is based on the Occupational Sitting and Physical Activity Questionnaire (OSPAQ), a validated instrument originally developed and tested in the Australian context. Its reliability and validity in assessing workplace sedentary time have been supported in previous studies (40). We adapted this variable by restricting its scope to the academic context of students´ life. The structure and response options of the original items were retained, only the context was adapted from an occupational to an academic setting.

#### Outcome 2: sitting duration during a 90-min teaching unit

This outcome variable reflects the self-reported duration of sitting during a 90-min teaching unit. Participants were asked: “On average, how many minutes of a 90-min teaching unit do you spend sitting?” The distribution of sitting duration was highly skewed, with a strong ceiling effect near 90 min. Therefore, responses were categorized into two groups based on a median split: (1) more than 85 up to 90 min and (2) 0–85 min. The conceptual structure of this measure reflects approaches used in instruments such as the OSPAQ, which has shown acceptable validity (Spearman’s *r* ≈ 0.53) and high test–retest reliability (ICC = 0.84) for self-reported sitting durations across structured time periods (40). The structure and response options of the original item were not adapted.

#### Outcome 3: number of breaks from sitting during a 90-min teaching unit

This outcome variable captured the number of self-reported breaks from sitting during a standard 90-min teaching unit. Participants were asked: “On average, how often do you interrupt your sitting during a 90-min teaching unit?” Responses were categorized into two groups: (1) no interruptions and (2) one or more interruptions. This item aligns conceptually with the Workplace Sitting Breaks Questionnaire (SITBRQ), which has demonstrated good test–retest reliability for break frequency (Spearman’s *r* = 0.71) and acceptable classification agreement (Cohen’s *κ* = 0.74), supporting its use in assessing the frequency of sedentary interruptions in structured settings such as lectures (41). The structure and response options of the original item were not adapted.

### Independent variables

Table 1 presents an overview of all independent variables included in the study, along with their operational definitions, number of items, and the sources from which the respective measurement instruments were originally derived. All items were piloted in a pretest.

Whether a construct was assessed by more than one item, scales were operationalized by combining items representing the same construct. Items were only retained if they showed strong intercorrelations and improved internal consistency. A Cronbach’s alpha value of 0.70 or higher was considered acceptable for confirming the reliability of the scales (42). For exploratory purposes, a Cronbach’s alpha of ≥ 0.60 is also acceptable (43, 44). All socio-ecological levels and composite variables were treated equally in the analyses, without applying any weighting between or within levels.

### Covariates

The following variables were included as potential confounders in all regression models: age (in years), sex (female/male/diverse), and semester of study. Covariates were selected *a priori* based on theoretical rationale and previous evidence (21) linking these variables to sitting among university students, rather than on statistical pre-testing. Covariates (age, sex, semester of study and meeting WHO PA recommendations) were added in the final adjustment step to control for potential confounding.

### PA assessment

Participants were asked about their PA behavior using the item: “During the past 7 days, how often did you engage in the following physical activities?” For each activity, participants indicated the typical duration (in minutes) and intensity (light, moderate, vigorous). Weekly totals were calculated by multiplying reported frequency by duration and classifying activities as moderate or vigorous according to WHO criteria. Based on their responses, it was assessed whether they met the current WHO PA recommendations for adults (at least 150 min of moderate or 75 min of vigorous activity per week). Responses were coded as “yes” or “no.” The item was adapted from the validated single-item PA measure, which has been shown to reliably estimate whether individuals meet recommended PA guidelines (45).

### Missing values and imputation

Incomplete cases were identified based on item-level missingness following recommendations regarding data quality concerns for cases with extensive missingness (46). From the initial 822 participants, 139 were excluded due to complete non-response, most likely resulting from technical dropouts or participants who opened but did not proceed with the questionnaire. Participants with more than 59% missing responses were excluded from the analysis (*n* = 254). Missing data were imputed only for independent variables. Outliers were examined and addressed as part of preliminary data cleaning procedures. After data cleaning, approximately 11% of the values were missing, with missing data distributed relatively evenly across the dataset. Due to the extent of missing data, multiple imputation was applied to preserve statistical power and reduce bias in the regression models. The imputation was conducted using the Predictive Mean Matching (PMM) algorithm, which is well-suited for handling skewed distributions and helps maintain the original data structure (47, 48). A total of 11 imputations were performed to reflect the proportion of missing data in the dataset (49). The quality of the imputations was evaluated by comparing the distribution of the imputed values with the observed values.

### Statistical methods

Data processing was conducted using SPSS 30.0. Bivariate analyses were performed to explore the associations between individual, social and environmental variables and the three outcome variables of sitting. For outcomes 2 and 3, cases with missing data were retained by modeling missingness as a distinct outcome category to identify potential correlates of non-response and minimize systematic bias. As associations in these groups were comparable to those in the main analysis, results are not presented. Variables, such as age and metric measures of wellbeing, were treated as continuous in the regression models. To assess the unique contribution of each level of the socio-ecological model, block-wise regression models were conducted. Groups of independent variables were entered separately based on their theoretical level (individual, social, environmental), facilitating the examination of the influence of each level by not including the effects of the other levels in the model, allowing for the isolated examination of each level’s impact (50). Finally, fully adjusted models were run. This allowed for a comprehensive understanding of the determinants of sitting, with all statistical tests conducted at a two-sided significance level of 5% (*p* < 0.05). As all outcome variables were binary, logistic regression models were applied. For ease of interpretation, regression coefficients were exponentiated and reported as odds ratios (ORs) with corresponding 95% confidence intervals. Age, sex, semester of study and adherence to WHO PA recommendations were included as covariates in all multivariate models to control for potential confounding. These variables were additionally examined in bivariate analyses to explore simple associations with sitting outcomes. However, as covariates, their coefficients are not displayed in the multivariate tables. To ensure model stability and interpretability in subsequent regression analyses, multicollinearity diagnostics were conducted. Items exhibiting high collinearity (e.g., variance inflation factor > 5) would have been excluded from the regression models, however, no such collinearity was observed among the independent variables included in the final models.

## Results

Descriptive statistics for key variables were presented as means and percentages for demographic and dependent variables (Table 2). The sample consisted of 434 students, with a mean age of 22.9 ± 3.5 years (age range: 19–47 years). Female participants represented 81.8% of the sample, reflecting the predominantly female enrollment in the selected study programs. In terms of semester level, 29% were in their 1st or 2nd semester, with a further distribution across semesters 3–10 (between 13 and 16%). On average, students spent 75.6% of the academic day being sedentary. More than three quarters of the participants reported sitting for a percentage share of 61–100% of the academic day. During a typical 90-min teaching unit, 37.8% of respondents reported no breaks from sitting and 83.8% reported sitting for more than 60 min continuously during these lectures, with 62.2% of them remaining seated for the entire 90-min teaching unit. Approximately 60% of the participants did not meet WHO PA recommendations.

**Table 2:** Descriptives of the sample and the outcome variables.

	Sample size (n)^1^	Percentage (%)	Mean (M)	Standard deviation (SD)
Age	401		22.9	3.5
Sex				
Female	355	82.8		
Male	70	16.3		
Diverse	4	0.9		
Level of study			5.4	3.6
1st or 2nd semester	125	29.8		
3rd or 4th semester	69	16.6		
5th or 6th semester	71	17.1		
7th or 8th semester	60	14.4		
9th or 10th semester	58	13.9		
More than 10 semesters	34	8.2		
Percentage of sedentary time during the academic day*			75.6	16.7
0–80%	273	63.1		
80.1–100%	160	36.9		
Hours spent sitting during the academic day**	421		12.4	3.2
Total sedentary time during a 90-min teaching unit			85.1	11.0
0–30 min	8	1.0		
31- < 60 min	425	99.0		
Number of breaks from sitting during a 90-min teaching unit			0.8	1.2
0	164	55.2		
≥1	133	44.8		
Meeting WHO recommendations				
Yes	260	16.8		
No	73	59.9		
Missings	101	23.3		

M, Mean; SD, Standard deviation; WHO, World Health Organization. ^1^Due to missing data, the descriptive results are based on different sample sizes. For the regression analysis, the imputed sample was used. *Assessed using the question: “What percentage of your academic day did you spend sitting, standing, walking/using a desk bike, or engaging in vigorous physical activity? (This refers only to your university-related activities and does not include commuting to or from campus or your leisure time.).” **Assessed using the question: “Please estimate how much time you spend sitting in the following situations each day, in minutes or, if applicable, hours.” Predefined situations were: commuting, on campus, studying, leisure screen time and non-screen leisure.

### Reported sedentary times and breaks from sitting in daily university life

On average, students self-reported sitting for 12.4 h per day, corresponding to 75.6 ± 16.7% of their academic day. During a standard 90-min teaching unit, students interrupted their sitting 0.8 ± 1.2 times on average. The mean sitting duration per 90-min teaching unit was 85.1 ± 11 min. The distribution of sitting showed that 39.5% of students spent between 61 and 80.9% of their academic day sitting, while 36.9% reported sitting for more than 80.9% of their academic day. Regarding sitting interruptions, more than half of students (55.2%) reported no breaks during a typical 90-min teaching unit. Furthermore, the majority of students (98.1%) reported sitting for 60 min or more during a 90-min teaching unit.

### Bivariate analysis—socio-ecological correlates of sitting

Findings of the bivariate analysis are depicted in Table 3. At the individual level, several variables were significantly associated with sitting. Students with higher motivational behavior specific self-efficacy, that is, a stronger belief in their ability to change their sitting, had statistically higher odds of belonging to the group that spent less than 80% of the academic day sitting (OR = 1.34, 95% CI: 1.04–1.74). They were also significantly more likely to report sitting for less than 85 min during a 90-min teaching unit (OR = 2.48, 95% CI: 1.84–3.33). Additionally, they had higher odds of reporting at least one break from sitting during a 90-min teaching unit (OR = 2.32, 95% CI: 1.67–3.22). Similarly, greater autonomy was significantly associated with higher odds of lower sitting across all three outcomes.

**Table 3:** Bivariate associations of socio-ecological correlates with sitting (ORs and 95%-CIs).

Socio ecological level	Variable	DV1 (daily sedentary time in %)	DV2 (sedentary time within 90 min)	DV3 (no. of breaks)
		OR (CI)*	OR (CI)**	OR (CI)***
Individual	Age	1.00 (0.94–1.06)	**1.08 (1.01–1.15)**	1.03 (0.96–1.10)
	Sex (female)	0.93 (0.55–1.55)	0.95 (0.56–1.60)	0.85 (0.47–1.52)
	Semester of study	0.94 (0.90–1.00)	0.96 (0.90–1.02)	0.99 (0.93–1.06)
	PA recommendation	0.74 (0.44–1.25)	1.53 (0.85–2.75)	1.51 (0.80–2.87)
	Habitual sedentary behavior	0.88 (0.72–1.08)	**0.79 (0.63–0.99)**	0.85 (0.66–1.09)
	Cue-dependent breaks	**0.83 (0.70–0.99)**	0.91 (0.75–1.10)	0.84 (0.67–1.04)
	Mental state	1.18 (0.91–1.54)	0.93 (0.69–1.25)	0.85 (0.60–1.19)
	Physical wellbeing	0.88 (0.68–1.14)	1.31 (0.98–1.74)	1.27 (0.92–1.75)
	Autonomy	**1.22 (1.03–1.43)**	**1.68 (1.39–2.03)**	**1.52 (1.24–1.85)**
	Motivational self-efficacy	**1.34 (1.04–1.74)**	**2.48 (1.84–3.33)**	**2.32 (1.67–3.22)**
	Attitude	1.03 (0.89–1.19)	1.10 (0.94–1.28)	**1.29 (1.09–1.53)**
Social	Perceived social norm	**1.40 (1.16–1.69)**	**2.19 (1.73–2.77)**	**1.83 (1.44–2.32)**
	Subjective norm	1.04 (0.89–1.22)	**0.76 (0.64–0.91)**	**0.72 (0.59–0.87)**
Physical environment	Physical environment	1.32 (0.89–1.96)	**3.31 (2.10–5.21)**	**3.43 (1.98–5.95)**

DV, Dependent variable; OR, Odds ratio; CI, Confidence interval. *Reference group = participants reporting more than 80% sedentary time. **Reference group = participants reporting 85–90 min sedentary time in 90 min. ***Reference group = participants reporting no interruptions in 90 min. Bold values indicate statistically significant associations (*p* < 0.05).

At the social level, perceived social norms, representing broader cultural support for standing and movement within the academic environment, were significantly associated with all three sitting outcomes. Higher perceived social norms for standing up were linked to significantly higher odds of spending less than 80% of the academic day sitting (OR = 1.40, 95% CI: 1.16–1.69), higher odds of not sitting through an entire 90-min teaching unit (OR = 2.19, 95% CI: 1.73–2.77) and higher odds of taking at least one sitting break (OR = 1.83, 95% CI: 1.44–2.32). Subjective norms, reflecting perceived social expectations regarding one’s own sitting, were also significantly associated with two outcomes: students reporting stronger subjective norms for sitting had lower odds of not sitting through the full lecture (OR = 0.76, 95% CI: 0.64–0.91) and of taking any sitting breaks (OR = 0.72, 95% CI: 0.59–0.87).

At the environmental level, perceiving the physical environment as supportive of being physically active was significantly associated with both lecture-based outcomes. Students who perceived their environment as more conducive to PA had significantly higher odds of not sitting through the entire 90-min teaching unit (OR = 3.31, 95% CI: 2.10–5.21) and higher odds of taking at least one sitting break during that period (OR = 3.43, 95% CI: 1.98–5.95).

### Multivariate models of socio-ecological variables

Findings of the multivariate analysis are depicted in Table 4. In the multivariate models, the influence of each socio-ecological level was first examined separately using blockwise logistic regression (model A), followed by a fully adjusted model including all variables of all levels simultaneously (model B). At the individual level, motivational self-efficacy is the most consistent correlate across all three outcomes. For spending less than 80% of the academic day sitting, a significant association was observed only in the fully adjusted model (OR = 1.43, 95% CI: 1.02–2.05). Mental state was significantly associated with reduced sedentary time in both models (blockwise: OR = 1.47, 95% CI: 1.05–2.05; fully adjusted: OR = 1.60, 95% CI: 1.07–2.42), but not with lecture sedentary time or breaks. Physical wellbeing was significantly related to lower overall sedentary time (blockwise: OR = 0.71, 95% CI: 0.52–0.97), but this association was not maintained in the fully adjusted model and no significant associations were found with the other outcomes. Autonomy was significantly related to both lecture sedentary time (OR = 1.40, 95% CI: 1.14–1.73) and breaks from sitting (OR = 1.28, 95% CI: 1.02–1.60) in the blockwise models.

**Table 4:** Multivariate associations of socio-ecological correlates with sitting (ORs and 95%-CIs).

Socio ecological level	Independent variable	DV1 blockwise**** ^A^	DV1 fully adjusted ******^B^	DV2 blockwise ^A^	DV2 fully adjusted ^B^	DV3 blockwise ^A^	DV3 fully adjusted ^B^
		OR (CI)*	OR (CI)*	OR (CI)**	OR (CI)**	OR (CI)***	OR (CI)***
Individual	Habitual sedentary behavior	0.92 (0.74–1.15)	0.95 (0.74–1.22)	0.78 (0.59–1.04)	0.83 (0.61–1.12)	0.90 (0.67–1.21)	0.90 (0.65–1.24)
	Cue-dependent breaks	0.88 (0.72–1.07)	0.90 (0.72–1.13)	1.12 (0.87–1.50)	1.23 (0.93–1.62)	0.99 (0.75–1.29)	1.03 (0.77–1.36)
	Mental state	**1.47 (1.05–2.05)**	**1.60 (1.07–2.42)**	0.81 (0.55–1.20)	0.72 (0.45–1.17)	0.81 (0.53–1.24)	0.78 (0.47–1.31)
	Physical wellbeing	**0.71 (0.52–0.97)**	0.70 (0.48–1.02)	**1.46 (1.01–2.10)**	1.26 (0.81–1.96)	1.35 (0.91–2.02)	1.26 (0.79–2.03)
	Autonomy	1.14 (0.94–1.37)	0.90 (0.71–1.14)	**1.40 (1.14–1.73)**	1.04 (0.80–1.36)	**1.28 (1.02–1.60)**	1.01 (0.76–1.35)
	Motivational self-efficacy	1.24 (0.93–1.65)	**1.43 (1.02–2.05)**	**2.12 (1.52–2.95)**	**2.04 (1.32–3.16)**	**2.00 (1.38–2.90)**	**2.10 (1.34–3.30)**
	Attitude	1.06 (0.91–1.24)	1.17 (0.97–1.42)	1.07 (0.89–1.28)	0.98 (0.77–1.24)	**1.26 (1.03–1.54)**	1.12 (0.88–1.42)
Nagelkerke *R*^2^		0.063		0.221		0.231	
Social	Perceived social norm	**1.43 (1.18–1.73)**	1.33 (1.00–1.77)	**2.15 (1.70–2.72)**	**1.56 (1.12–2.19)**	**1.77 (1.39–2.26)**	1.30 (0.90–1.89)
	Subjective norm	1.10 (0.94–1.30)	**1.26 (1.01–1.56)**	**0.82 (0.67–0.996)**	0.93 (0.71–1.20)	**0.75 (0.61–0.93)**	0.86 (0.66–1.13)
Nagelkerke *R*^2^		0.052		0.171		0.118	
Physical environment	Perceived physical environment	1.33 (0.91–1.95)	1.05 (0.60–1.82)	**3.31 (2.10–5.21)**	**2.18 (1.23–4.22)**	**3.43 (1.98–5.95)**	**2.14 (1.04–4.40)**
Nagelkerke *R*^2^		0.009	0.123	0.096	0.347	0.081	0.355

DV, Dependent variable; OR, Odds ratio; CI, Confidence interval. ^A^The influence of each level was calculated separately, adjusting for all other variables within the level of influence. ^B^The influence of all three levels was calculated simultaneously, adjusting for all other variables independent of the level. *Reference group = participants reporting more than 80% sedentary time. **Reference group = participants reporting 85–90 min sedentary time in 90 min. ***Reference group = participants reporting no interruptions in 90 min. ****Adjusted for co-variates (age, sex, semester of study, WHO recommendations met). *****Adjusted for co-variates + all other variables. Bold values indicate statistically significant associations (*p* < 0.05).

At the social level, perceived social norms were significantly associated with all three outcomes in the blockwise models and remained significant in Outcome 2 after adjustment (OR = 1.56, 95% CI: 1.12–2.19), respectively. This shows that perceived social norms for standing and moving reduced the odds of being more sedentary. Subjective norms were significantly associated with lecture sedentary time and sitting breaks in the blockwise models but not in the fully adjusted models.

At the environmental level, blockwise models showed that perceiving the physical environment as more activity-permissive was associated with significantly higher odds of not sitting through the full 90-min teaching unit (OR = 3.36, 95% CI: 2.10–5.38) and with higher odds of taking at least one sitting break (OR = 3.28, 95% CI: 1.91–5.65). These associations remained statistically significant in the fully adjusted models. The explanatory power of the socio-ecological models varied considerably across outcomes (Table 4). The fully adjusted models explained the most variance, particularly for lecture-related behaviors (*R*^2^ = 0.347 and *R*^2^ = 0.355), while overall sedentary time showed lower explained variance (*R*^2^ = 0.123).

## Discussion

To the best of our knowledge, the present study is one of the first to examine specific time segments of sitting during an academic day and shows that university students are highly sedentary during their academic course of the day in general and especially during teaching units. During a standard 90-min teaching unit, students remained seated for approximately 85 out of 90 min (94.4% of the lecture time), with minimal interruptions of sitting. Notably, 55.2% of students did not interrupt their sitting at all. Following the levels of a socio-ecological approach to explore the correlates of sitting in university students we found that motivational self-efficacy emerged as a key correlate at the individual level, associated with reduced sitting and more frequent breaks from sitting. Movement-supportive social norms were associated with reduced sedentary time, while subjective norms reinforcing sitting acted as behavioral barriers at the social level. Finally, perceived physical environments that afford and encourage being physically active contributed to less prolonged sitting at the environmental level.

Our results are in line with others and confirm the high prevalence of sitting in this target group and highlight the role of social and environmental variables in shaping sitting in educational contexts (10, 20, 22, 29, 35, 37). This underlines the rigidity of lecture-based learning environments, in which opportunities for movement are structurally constrained. Fixed seating arrangements, long periods of scheduled immobility, and the lack of flexible learning spaces limit students’ ability to vary posture or interrupt sedentary time (36, 37). The high prevalence of uninterrupted sitting observed in our data also highlights the extent to which the formal structure of academic sessions contributes to physical passivity, independent of individual motivation. As such, academic teaching formats can be considered a structurally sedentary context and may constitute a critical point of intervention for health-promoting behavioral change.

Our study also reveals the excessive sitting among university students and underscores that it is shaped by interacting individual, social and environmental factors. Interventions aiming to reduce prolonged sitting should therefore target all three socio-ecological levels simultaneously. While promoting PA remains important, it is not sufficient to address SB per se, as individuals can meet PA recommendations while still accumulating excessive sedentary time. Reducing uninterrupted sitting requires specific strategies such as encouraging regular breaks, providing activity-supporting environments and fostering supportive social norms within academic settings.

### Correlates of overall and domain-specific sedentary time

At the individual level, motivational self-efficacy emerged as a consistent predictor of lower sitting and more frequent sitting interruptions. This supports theoretical models such as the Health Action Process Approach (33), which posit that belief in one’s capacity to act is central to behavioral regulation. Our findings also echo university-specific research by Castro et al. (8), who showed that stronger self-efficacy predicts students’ intentions to reduce sitting. However, motivational resources alone may be insufficient when students experience low behavioral autonomy due to rigid instructional structures. Particularly in academic settings with traditional teaching formats, like the small-group seminars in our study, students’ opportunities to act on self-regulatory intentions are often constrained by implicit role expectations and lecturer-controlled pacing (29, 51). This structural limitation may partially undermine the actualization of behavior-specific self-efficacy beliefs in everyday behavior. To effectively strengthen self-efficacy, interventions should address its multiple psychological roots, including opportunities for successful behavioral experiences, positive role models, verbal encouragement, and supportive emotional climates (52). Such approaches can be embedded in academic settings through small peer-based coaching programs, lecturer-initiated prompts to move, or curricula that explicitly promote autonomy-supportive environments (51). These measures not only enhance individual capacity for behavioral regulation but may also initiate broader cultural shifts toward more active academic norms—especially since self-efficacy becomes most relevant when individuals are required to overcome perceived barriers. Additionally, mental state showed a positive association with overall sedentary time, which appears counterintuitive. A likely explanation lies in a mismatch between the outcome and the operationalization of this independent variable (53). The mental state items were context-specific and referred exclusively to students’ experiences during academic sessions (e.g., “What effects do you notice after sitting for longer than 60 min during lectures?”). However, the outcome variable captured total sitting during the academic day. This misalignment may have led to measurement error, as this variable was not sufficiently representative of the broader behavioral context. Consequently, the observed association should be interpreted with caution and further research is warranted on this issue.

At the social (interpersonal) level, perceived social norms consistently showed significant associations with sitting across multiple outcomes. Stronger norms supporting movement were linked to lower sedentary time and more frequent interruptions of sitting, whereas subjective norms reinforcing sitting were associated with greater sitting. These findings align with previous research identifying social expectations as key determinants of health-related behaviors in academic settings (23, 28). In contexts where sitting is perceived as the default posture, supportive norms can provide behavioral permission to engage in movement. This underscores the relevance of shaping a shared culture that legitimizes posture variation and reduces sitting within university environments. Power dynamics within academic settings may further influence these relationships, as lecturers set behavioral expectations and regulate opportunities for movement (37). Cross-level interactions between factors, such as motivational self-efficacy and interpersonal norms, may determine whether students feel authorized and capable of interrupting their sitting.

Overall, these findings highlight the critical role of the social environment in shaping students’ sitting. Interventions aimed at reducing sedentary time in academic settings should therefore not only focus on individual motivation but also explicitly address the social and normative climate. Beyond the immediate academic context, these social and normative climates are embedded within broader systemic and cultural norms that define acceptable behavior in higher education. Prevailing academic conventions often equate attentiveness and professionalism with stillness, reinforcing sitting as the default posture for learning (37). Moreover, institutional structures that prioritize efficiency, cognitive output and tightly scheduled teaching formats may inadvertently discourage movement and normalize sitting (21, 37, 54). Addressing sedentary culture in universities therefore requires not only local behavioral interventions but also broader institutional and cultural reflection on how learning, performance and embodiment are valued in academic life (54). Strategies could include making movement-friendly norms more visible, encouraging instructors to model and support standing or movement and creating policies that normalize regular breaks of sitting within lectures and academic activities. The interaction between personal and interpersonal factors may further explain the social-normative patterns influencing students’ sitting. For instance, individual attributes such as motivational self-efficacy likely mediate the influence of social norms on sitting. Students with higher self-efficacy may be more responsive to supportive social cues and more capable of translating social approval for movement into action, whereas those with lower self-efficacy may perceive similar norms but lack the confidence to act on them (52). This highlights the importance of considering how personal resources interact with social influences when designing interventions to reduce sitting in academic settings.

The findings at the environmental level of our socio-ecological approach confirm the crucial role of environmental and institutional conditions in shaping students’ sitting during academic activities. Specifically, our results show that students who perceived their academic context as physically activating were more likely to interrupt sitting and less likely to remain seated throughout an entire teaching unit. This corresponds with previous studies demonstrating that perceived opportunities for movement in the physical and institutional environment play a central role in enabling less sitting (51, 55, 56). Such environments are typically characterized by architectural and organizational features, such as standing desks, flexible seating or modular classroom layouts, that facilitate behavioral alternatives to sitting (51, 55). Conversely, traditional passive teaching formats and static room arrangements have been shown to reinforce prolonged sitting and discourage movement (10, 29).

While environmental features have received increasing attention, the role of teaching staff in shaping physical and social affordances in academic settings remains largely overlooked in current research (29, 35). Our findings show that students remain seated for approximately 85 out of 90 min during typical lectures, with minimal interruptions, a pattern that underscores the structural rigidity of academic instruction formats and highlights the university classroom as a predominantly sedentary environment (36, 37). These sedentary patterns reflect not only environmental or individual factors, but also the implicit behavioral expectations set by instructors. Lecturers influence student behavior both directly, through instructional design, and indirectly, through modeling and permissiveness toward movement (29). Despite growing awareness of the health risks associated with prolonged sitting, movement-friendly teaching practices remain rare. Efforts to reduce sitting should therefore not only address physical space but also actively support teaching staff in legitimizing and implementing posture variation and activity during academic sessions.

### Explained variance of socio-ecological model (R^2^)

The comparison of bivariate, blockwise and fully adjusted logistic regression models demonstrated that the inclusion of covariates did not materially alter the associations between key socio-ecological correlates and sitting, indicating that these effects were robust to statistical adjustment. Covariates (age, sex, semester of study and meeting WHO PA recommendations) were incorporated only in the final adjustment step to control for potential confounding. The stepwise increase in explained variance (Nagelkerke R^2^) across individual, social and environmental levels further underscores that sitting is influenced by multiple interacting correlates, reinforcing the relevance of a socio-ecological perspective. Meeting the WHO PA recommendations, included *a priori* as a confounder, did not materially influence the observed associations, and other covariates likewise showed no substantive impact.

From a socio-ecological perspective, our findings and reflections about academic sitting in university students underscore how sitting in academic settings results from the interplay of individual regulation and context-specific affordances. Prior research has shown that students often feel ‘forced to sit’ due to a lack of perceived alternatives (10), suggesting that behavior change interventions must target not only awareness and motivation but also the structural and normative conditions that shape student behavior. Taken together, the findings emphasize the importance of integrating physical, pedagogical and normative aspects of the university environment when aiming to reduce sitting in higher education settings. Beyond the institutional context, sitting in higher education also reflects broader cultural and systemic structures that normalize sitting as the default posture in academic and professional life (14). Universities function as formative social spaces where behavioral norms are learned and reproduced, shaping expectations that extend into workplace and societal contexts. From a macro-level perspective, reducing prolonged sitting in higher education may therefore contribute to shifting wider cultural norms around movement and work, with potential long-term implications for population health, productivity and healthcare burden (14, 57). Recognizing sitting as a systemic issue highlights the need for coordinated policy action and sector-wide frameworks that support active learning cultures beyond individual institutions.

### Strengths and limitations

This study offers several notable strengths. Most importantly, it is based on a large student sample, which enabled robust statistical analyses and increased the reliability of observed associations. In addition, it provides a uniquely specific though comprehensive view of sitting in university settings by simultaneously capturing behavioral patterns, such as uninterrupted sedentary time during teaching units and a broad spectrum of individual, social and environmental determinants. This socio-ecological framework allows for a more differentiated understanding of sitting, which remains underrepresented in research on university populations (56, 58). Moreover, the study covered sedentary patterns across the entire academic course of the day.

At the same time, several limitations must be kept in mind when interpreting our findings. First, the study employed a cross-sectional design and relied on self-reported data, which limits causal inference. Recall bias and social desirability may have influenced the accuracy of responses (59, 60). Second, the questionnaire was relatively long, as it was designed to collect data for two separate research projects. This may have led to participant fatigue or reduced data quality and likely contributed to the number of missing responses. Selection bias may also be present, as students with greater health awareness may have been more likely to participate. Additionally, the skewed distribution of sitting, with many students already reporting high sedentary times, reduced the variance available for predictive modeling. While the socio-ecological framework guided variable selection, most predictors in this study were situated at the individual level. This imbalance partly reflects the state of the field, as validated measures for social and environmental determinants of sitting in academic contexts remain limited (60, 61). Consequently, the strength of individual-level associations may appear inflated relative to underrepresented contextual factors. Furthermore, it is worth noting that the study was conducted at a university with a long-standing institutional commitment to promoting PA, including previous interventions such as the “Kopf-Stehen” project and movement-supportive infrastructures (e.g., active learning spaces, standing tables, table tennis areas). The fact that substantial sitting was still observed under these conditions suggests that the situation may be even more pronounced at institutions without such a supportive environment. Finally, the sample was drawn from a single university with a predominance of female students, which may limit generalizability to other institutional or cultural contexts. Furthermore, the overrepresentation of female students precluded meaningful comparisons between male and female students, which should be acknowledged as a limitation. Nevertheless, the socio-demographic profile of the sample is broadly comparable to similar university populations, supporting cautious external validity. Moreover, as previously noted, a mismatch between context-specific predictor items (e.g., mental state during lectures) and general outcome measures (e.g., total sedentary time) may have introduced measurement error and affected construct validity (53).

## Conclusion

This study demonstrates that SB among university students is shaped by interacting individual, social and environmental factors. Motivational self-efficacy emerged as the most consistent determinant across all outcomes, while perceived social norms and the physical environment were particularly relevant for teaching unit-related SB. These findings underscore that interventions targeting SB in academic settings should address all three socio-ecological levels simultaneously. Focusing solely on individual motivation is unlikely to be sufficient without complementary structural and cultural support. Universities are therefore encouraged to consider active teaching formats, activity-supportive learning environments and institutional policies that normalize movement during academic sessions.

## Data Availability

The raw data supporting the conclusions of this article will be made available by the authors, without undue reservation.

## Supplementary material

Supplementary File 1 English translation of the self-reported questionnaire used in this study.
