# Supplementary material for: Sedentary behavior among university students and its socio-ecological correlates during the academic day: a cross-sectional study
Source: Front Public Health. 2026 Aug 17;14:1754818. doi: 10.3389/fpubh.2026.1754818 (PMC13527041; doi:10.3389/fpubh.2026.1754818)
Supplement: Supplementary file 1 [file Supplementary_File_1.docx]

**Please note:**

1. This version of the questionnaire is a plain text version translated from German to English since we applied a web-based tool.
2. Table 1 of our manuscript shows which established scales we used and adapted to our context, respectively.

**Sociodemographic information**

- How old are you? (years)
- What is your gender? (female / male / diverse)
- What is your current semester of study? (number)
- Are you enrolled in a Bachelor or Master program? (Bachelor / Master)
- What is your study program? (Secondary Education / Primary Education / Non-teaching degree programs / Special Education / No answer)
- Where do your courses mainly take place? (select all that apply)
  - Old building
  - New building
  - Sports facilities
  - Czernyring
  - Technology Park
  - Other (please specify)

**Sitting time in daily accademic life**

Please estimate the percentage of your study-related time (e.g., at university, in lectures, studying at home or in the library) spent on the following activities over the past 7 days. (Total must equal 100%)

- Sitting
- Standing
- Walking / Using a desk bike
- Vigorous physical activity

**Sedentary time in courses**

**Do you have practical classes this semester (e.g., sports, home economics, art, experiments)?** (Yes / No)

If yes:

- How often do you interrupt sitting during a 90-minute class? (Number)
- How many minutes of a 90-minute theoretical class do you spend sitting?
- How many minutes of a 90-minute practical class do you spend sitting?
- For what reasons do you interrupt your sitting time in class? (Open answer)

If no:

- How often do you interrupt sitting during a 90-minute class?
- How many minutes of a 90-minute theoretical class do you spend sitting?
- For what reasons do you interrupt your sitting time in class? (Open answer)

**Determinants influencing sedentary behavior**

(Responses on a 5-point Likert scale: strongly disagree – disagree – neutral – agree – strongly agree)

**Attitudes**

- I believe prolonged, uninterrupted sitting negatively affects my health.
- I believe a dynamic teaching and learning environment (with less sitting and more movement) positively affects my attention.
- I want to sit less in classes.

**Autonomy**

- I am allowed to decide for myself when I stand up during class.
- I am allowed to decide for myself how much time I spend sitting during class.

**Habitual sitting**

Sitting is something I do in class without thinking about it.

**Cue-dependent breaks**

- I need a prompt/reminder to remember to interrupt sitting more often in class.

**Perceived social norms**

In the classes I attend, there is a culture that supports standing and movement.

- I worry that I might be perceived as inattentive if I sit less during classes.
- I worry that others might find it irritating if I sit less during classes.
- The instructors support students in deciding how much they sit, stand, or move during class.
- Among fellow students, there is a culture that supports standing and movement.

**Motivational self-efficacy**

- I am confident that I can regularly interrupt my sitting time during classes.
- I am confident that I can integrate more movement into my classes.
- I am confident that I can stand up more frequently in class even if fellow students find it irritating.
- I am confident that I can stand up more frequently in class even if lecturers find it irritating.
- I am confident that I can integrate more movement into my daily student routine.

**Perceived physical environment**

(On a 4-point scale: always – often – rarely – never)

- Do you have access to sit-stand furniture (e.g., standing desks, desk risers) during classes?
- Do you have access to movement-supportive furniture (e.g., desk bikes, wobble stools, balance boards, standing mats, gym balls) during classes?
- Do you think the size of your seminar rooms allows for active learning?
- Do you think the equipment of your seminar rooms allows for active learning?

**Mental state and physically well-being during classes**

(Responses on a 4-point Likert scale: applies – rather applies – rather does not apply – does not apply)

- Tension in the neck and shoulder area
- Back tension
- Knee pain
- Headaches
- Lack of legroom
- Inner restlessness
- Decreasing ability to concentrate
- Decreasing motivation
- Decreasing ability to memorize
- Increasing fatigue
- Other effects (please specify)

**Daily sitting time estimation**

Please estimate how much time per day (in hours/minutes) you spend sitting in the following situations:

- On a typical weekday
- On a typical weekend day
- While traveling (e.g., car, bus, visiting friends, cinema, supermarket)
- At the university (e.g., library, PC pool, cafeteria, lecture hall, seminar room)
- Studying at home or other places
- Screen time during leisure (e.g., PC, TV, gaming, tablet, smartphone)
- During leisure time without screen use (e.g., visiting friends, theater, dining out, reading)

**Physical activity**

How often did you perform the following activities in the past 7 days? (Number of times + average duration in minutes)

- Vigorous physical activity (increased effort and sweating, e.g., intensive swimming, jogging, fitness workout, cycling)
- Moderate physical activity (low effort and light sweating, e.g., brisk walking, slow cycling, slow swimming, yoga)
- Light physical activity (no effort, no sweating, e.g., slow walking, fishing)
